# Amorphous Nanoparticulate Formulation of Sirolimus and Its Tablets

**DOI:** 10.3390/pharmaceutics10030155

**Published:** 2018-09-11

**Authors:** Yudong Shen, Xingya Li, Yuan Le

**Affiliations:** State Key Laboratory of Organic-Inorganic Composites, Beijing University of Chemical Technology, Beijing 100029, China; 2015210017@mail.buct.edu.cn (Y.S.); 2016200042@mail.buct.edu.cn (X.L.)

**Keywords:** amorphous sirolimus nanoparticles, antisolvent precipitation, high gravity rotating packed bed, dissolution rate, tablet formula

## Abstract

Nanocrystallization and amorphization have proven to be two effective strategies to improve the bioavailability of water-insoluble drugs. The purpose of our work was to develop a nano-formulated tablet of sirolimus (SRL) for enhanced dissolution. Amorphous SRL nanocomposites were prepared using anti-solvent precipitation via a high-gravity rotating packed bed. Various factors that affect particle size and size distribution, such as excipients, rotating speed, antisolvent/solvent flow rate, were investigated. Structure, stability and in vitro dissolution of the as-prepared SRL were evaluated. Furthermore, the nanoparticulated SRL tablet formula was screened to control drug release. Importantly, SRL tablets exhibit different dissolution profile by adjusting HPMC (hydroxypropyl methyl cellulose) content, which makes them more suitable for various formulation developments.

## 1. Introduction

In recent years, administering oral medications has been the most extensive and convenient route for treatment of various diseases [1]. However, about 40% of the new chemical entity drugs are insoluble in water, which usually leads to low oral bioavailability and poses a huge challenge for new drug development [2]. Consequently, increasing the solubility of drugs in water to improve their oral bioavailability is a sticking point in the development of novel pharmaceutical formulations. Lots of formulation strategies have been presented to solve these problems, including self-emulsifying formulations, ionic inclusion complexation, solid dispersions, lipid-based complexation, and nanoparticulate formulation [3].

To meet the growing need for nanopharmaceutical formulation development, nanoparticles are generally produced by breaking down large particles using a relatively simple and efficient top-down process. Two of the typical means are wet ball milling and high-pressure homogenization [4,5]. The advantages of top-down processes in industrialized production has made them successful for some marketed drugs such as Megace^®^ ES (megesterol acetate), Tricor^®^ (fenofibrate), Invega Sustenna^®^ (paliperidone palmitate) and Rapamune^®^ (sirolimus) [6].

Sirolimus (SRL) is a new type of macrocyclic lactone immunosuppressant, one possessing a high activity on anti-rejection reaction after organ transplantation. Its mechanism of action is completely different from traditional immunosuppressants such as cyclosporine and tacrolimus. SRL can form an immunosuppressive complex with the immunophilin FKBP-12 (FK506-binding protein 12), inhibiting mTOR (mammalian target of rapamycin) activity, thereby blocking signaling that activates the immune system and rejection [7].

Due to its high permeability and poor water solubility, SRL is classified as belonging to the BCS (biopharmaceutics classification system) class II drug category [8]. It possesses a very low aqueous solubility, about 2.6 μg/mL [9]. The poor water solubility and low dissolution rate reduce its absorption, resulting in low bioavailability [10]. The original oral administration of SRL was through a lipid-based liquid solution with a bioavailability of 14%. With the application of wet-ground in Nanocrystal^®^ technology, SRL is ground in a ball mill for 120 h to prepare nanosized drug dispersions, and then post-processed into a tablet formulation as Rapamune^®^ [6,11]. The bioavailability of nanotablets is increased by approximately 27%, while having no storage limitations and improved patient compliance [6,12,13].

Compared with top-down commercial technology, the bottom-up process involving the assembly and control of precipitations of nanoscale drug particles has been attracting more attention. It provides a completely new path to control particle morphology/crystals, as well as to tailor nanoparticle surface functionality and to combine multiple APIs in a single nanocarrier [14]. However, as we know, there has been no commercial product produced by bottom-up methods. It is a strong innovation to develop general techniques for the stable preparation of pharmaceutical nanoparticles to achieve the potential for nanoparticle formulation.

High-gravity antisolvent precipitation (HGAP) is considered to be a promising technology due to its uninterrupted mass production and conduciveness to scaling-up in order to achieve industrialization [15,16]. A rotating packed bed (RPB) is generally employed to execute the HGAP process, and RPB can create a high-gravity environment in the bed to achieve intense mixing and mass transfer by rotating at high speed. Compared with the stirred tank, the mass transfer processes and micromixing efficiency in the RPB are greatly enhanced, which facilitates the system’s generation of a more homogeneous regional concentration and higher supersaturation, thereby achieving a rapid and uniform nucleation rate [17]. Therefore, the size and size distribution of the generated particles can be well controlled. HGAP technology has been successfully applied to the preparation of nanosized amorphous drugs such as itraconazole [18], cefuroxime axetil [19], and glibenclamide [20].

The main objective of this work is to prepare SRL amorphous nanodispersions using HGAP and further develop its tablets formulation. SEM (Scanning electron microscopy), XRD (X-ray diffraction), FT-IR (Fourier Transform infrared spectroscopy), and contact angle as well as in vitro dissolution were employed to characterize the stability, structure and release performance of SRL nanoparticles. Meanwhile, the properties of SRL tablets, such as friability, mean weight, hardness and dissolution, were also evaluated and compared with commercial Rapamune^®^ tablets produced by wet-mill.

## 2. Experimental Section

### 2.1. Materials and Equipment

**Figure 1:**
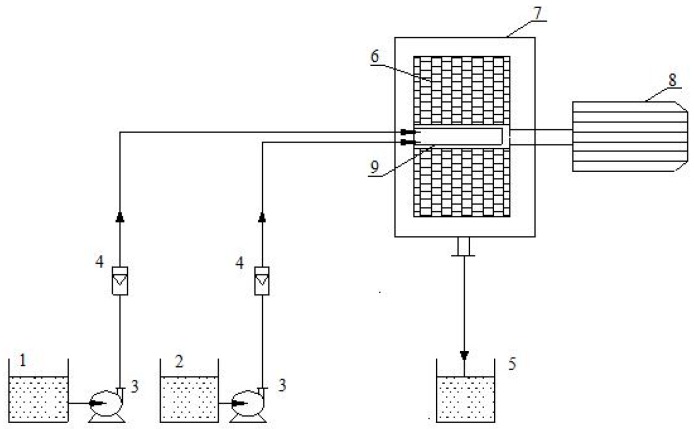
Schematic representation of HGAP process (1: drug solution tank; 2: anti-solvent tank; 3: pump; 4: flow meters; 5: tank; 6: packed rotator; 7: casing; 8: motor; 9: liquid distributor).

Raw SRL was purchased from Shanghai Nest Biological Engineering Co., Ltd. (Shanghai, China). Polyvinyl pyrrolidone (PVP), mannitol, sodium dodecyl sulfate (SDS), microcrystalline cellulose (MCC), magnesium stearate, hydroxypropyl methyl cellulose (HPMC) were supported by Beijing Chemical Factory (Beijing, China). Figure 1 shows the experimental device for the HGAP process. The core component of the RPB is a rotator that is wrapped in a wire mesh with an outer and inner diameter of 80 and 35 mm, respectively [21]. The liquid distributor consists of two stainless steel tubes with a wall thickness of 1.5 mm and an outer diameter of 7 mm. Part of the tubes extend into the rotator and have a slit with a width of 0.5–1.0 mm and a length of 10 mm. The solvent phase and the anti-solvent phase are pumped into the rotator through these slits from the two liquid storage tanks. The rotator is mounted in a fixed casing to remain stable during high speed rotation.

### 2.2. Preparation of SRL Nanoparticles

In a typical process, ethanol and deionized water were used as solvent and antisolvent, respectively. The quantitative raw SRL was weighed and dissolved in ethanol to prepare a desired concentration of drug solution, and the solution was filtrated to remove the insoluble impurities. Then, SRL drug solution and deionized water in which excipients were dissolved were added into tanks 1 and 2 kept at 20 °C, respectively. After that, the two liquids were pumped into the RPB through the distributors. These two phases were mixed in the packed rotator and SRL nanoparticles generated immediately due to changes in supersaturation. The obtained SRL suspension was collected and stored in tank 5. Finally, the obtained slurry was dried by a spray dryer (B-290, Buchi, Flawil, Switzerland) to get arid SRL powders under the following operating conditions: feed rate 5 mL/min, atomizing air rate 800 L/h, aspiration rate 36 m^3^/h, outlet and inlet temperatures of 80–90 and 160 °C, respectively. The as-formed SRL nano-powders were stored in a container protected from light at room temperature for subsequent testing.

### 2.3. Preparation of SRL Nanotablets

A flat-faced direct compression method was applied to prepare SRL nanotablets. Prior to compression, Mannitol, Microcrystalline cellulose (MCC), Hydroxypropyl methyl cellulose (HPMC), and Magnesium stearate used as excipients were mixed using a flexible mixer for 10 min at 40 rpm. Afterward, the spray-dried nanoparticles were mixed with excipients using a mixing machine. Tableting was performed under a compression force of 50–200 kN/cm^2^ using a single punch tableting machine. The prepared tablets had a mean weight of 316 mg, and the thickness of the nanotablets was about 2.5 mm. The dissolution rate of the prepared tablets was compared with that of the marketed tablets.

### 2.4. Characterization

#### 2.4.1. SRL Nanoparticles

SEM: Scanning electron microscopy (SEM) (Model S4800, Hitachi, Tokyo, Japan) was employed to observe the morphology of SRL samples. Slides with samples were attached to the sample stage and coated with gold under vacuum. Then, the sample stage was placed into the SEM sample chamber for observation. The size and size distribution of the nanoparticles were characterized by Zetasizer (Nano-ZS, Malvern, Britain).

FT-IR: The chemical composition and molecular structure of raw SRL and SRL nanoparticles were detected by FT-IR analysis. The samples were diluted with potassium bromide and compacted to obtain disks. FT-IR spectra were recorded using a Vertex 70 spectrometer (Bruker, Billerica, MA, USA) with a resolution of 2 cm^−1^ at room temperature in a wave number range of 4000–500 cm^−1^. 

XRD: X-ray diffraction (XRD) measurements were carried out to investigate the crystallinity and crystal form of raw SRL and SRL nanoparticles using a Model XRD-6000 diffractometer (Shimadzu, Kyoto, Japan). The sample was scanned at a scan speed of 5° per minute for a scan range of 5–60°.

Contact Angle: The solid water contact angle measurements were carried out using a wettability tester (OCA25, Dataphysics, Charlotte, CA, USA). 150 mg of raw SRL powers, SRL nanoparticles, physical mixtures (raw SRL powder/PVP/SDS) were pressed under a pressure of 10 MPa for 1 min respectively to prepare powder compacts. A droplet of about 2 mL in volume was impacted onto the powder compact using a microsyringe, followed by image capture and analysis to obtain the contact angle.

#### 2.4.2. SRL Nanotablets

Weight test: Six tablets from each batch were weighed using an electronic balance to calculate their mean weight, so as to ensure the uniformity of the tablets in each batch. The mean weight is expressed in mg (±SD) (Standard Deviation).

Hardness and friability: The hardness of six tablets from each batch was tested using a hardness tester. The mean hardness should be 30–50 N/cm^2^. A hardness tester was employed to measure the friability of tablets, six tablets from each batch were analyzed. After friability, the tablets were then dusted and reweighed.

Disintegration test: The disintegration test was carried out using a disintegration apparatus at 37.0 ± 5 °C in distilled water, six tablets from each batch were analyzed. The tablets were kept in the basket, which was lifted at a frequency of 30 rpm.

Dissolution test: A dissolution apparatus (Model D-800LS, TDTF, TianJin, China) was employed for dissolution test with the USP (United States Pharmacopoeia) Apparatus type-II method. A total volume of 900 mL phosphate buffer saline (PBS) solution with 0.5% SDS was used as the dissolution medium, which was kept at 37.0 ± 0.5 °C at 100 rpm. Raw SRL, physical mixed particles, nanosized SRL particles, SRL nanotablets and marketed tablets were transferred to 900 mL dissolution media, respectively. Aliquots (5 mL) were withdrawn at specific time intervals with a syringe filter, and replaced with an equal volume of fresh medium to keep the volume consistent. Samples were then analyzed with a UV (Ultraviolet Rays) spectrophotometer at 279.5 nm after being filtered through a syringe filter. Each sample was analyzed in triplicate.

## 3. Results and Discussion

### 3.1. Liquid Precipitation Process in the RPB

Liquid phase precipitation can effectively reduce the particle size and narrow the size distribution by adjusting nucleation rate and growth kinetics. The growth rate of the crystal is inhibited at a high nucleation rate, which promotes the assembly of nanoparticles with a narrow size distribution. The nucleation kinetics of poorly water-soluble drug during the HGAP process were investigated in our previous study [22]. We found that excipients, high-gravity field and AS/S flow ratio were key factors in influencing nucleation rate, and hence affected particle size [22]. Therefore, in this work, we firstly screened excipients. Different types of pharmaceutical excipients generally recognized as safe (GRAS) were selected based on particle size, and the optimal combination and ratio was screened out to be the formulation [23].

**Figure 2:**
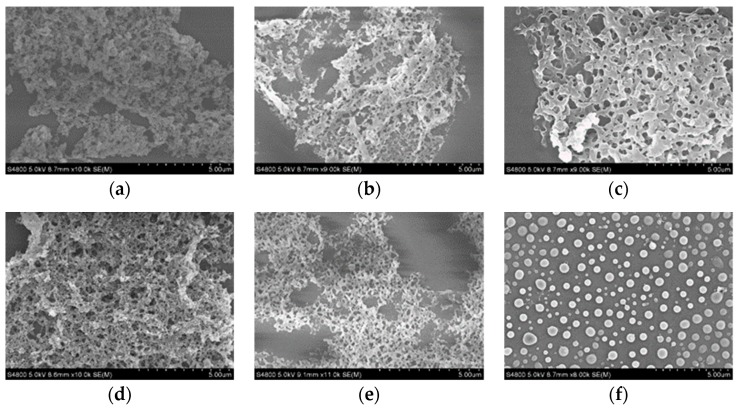
SEM images of (**a**) precipitated SRL without excipient; (**b**) HPMC; (**c**) HPMC with SDS; (**d**) Lactose; (**e**) PVP; (**f**) PVP-SDS.

Figure 2 shows SEM images of SRL precipitated with different excipients. The precipitated SRL show network-like morphology with sever aggregation even with excipients such as HPMC, HPMC-SDS, Lactose and PVP. However, when SRL was precipitated with PVP combined with SDS, the particles exhibited a separated spherical shape with a size of approximately 530 nm. The reason PVP-SDS can effectively prevent particles from agglomerating and stably dispersing is probably due to the interaction between PVP and SDS in solution. SDS can be clustered on the PVP molecular chain to form micelles or soft matter clusters, the micelles have the dual effects of electrostatic stabilization and steric hindrance, which can effectively reduce the surface free energy of the particles to a certain extent, preventing the particles from agglomerating, and exerting a better dispersion and stabilization effect [24].

**Figure 3:**
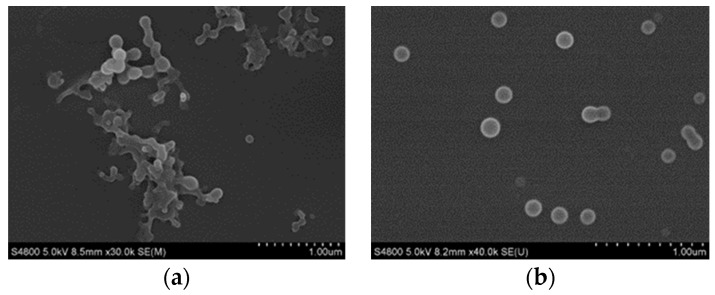

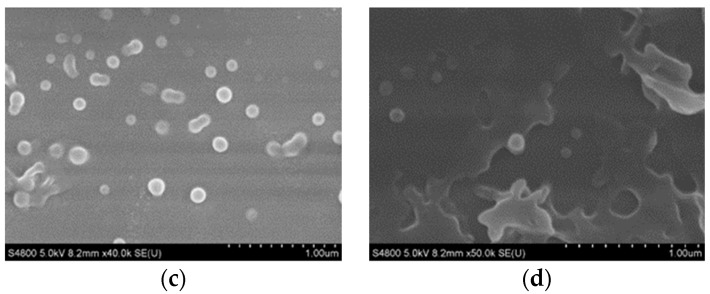
SEM images of SRL nanoparticles prepared at different PVP additions (**a**) 50%; (**b**) 100%; (**c**) 150%; (**d**) 200%.

Furthermore, the morphology and particle size of SRL were generally highly affected by the proportion of PVP, as shown in Figure 3. The amount of SDS was fixed at 5 wt. % of the drug, and then weight ratio of PVP and the drug was adjusted. When PVP:SRL = 0.5:1, the particles aggregated; enhancing PVP content to PVP:SRL = 1:1, the particles were well dispersed and the size was about 150 nm. When continuing to increase the amount of PVP to 1.5:1, the particle size still remained at 150 nm. When the ratio of PVP exceeded 2:1, the drug particles became larger and a sheet-like morphology appeared. This phenomenon could be explained by two aspects: (1) when the concentration of PVP was relatively low, it meant there was an insufficient amount of PVP molecules in the solution. Accordingly, only some of the PVP was adsorbed onto the surface of the nucleus to separate the drug particles, which caused the aggregation of particles; and (2) excess PVP resulted in a large number of PVP molecules in the solution, and the long chains of the PVP molecules would have entangled with each other when the concentration was high, causing the system to flocculate. Higher PVP concentration would increase the possibility of long chains of PVP molecules entangling with each other, causing the aggregation and enlargement of SRL drug particles.

**Figure 4:**
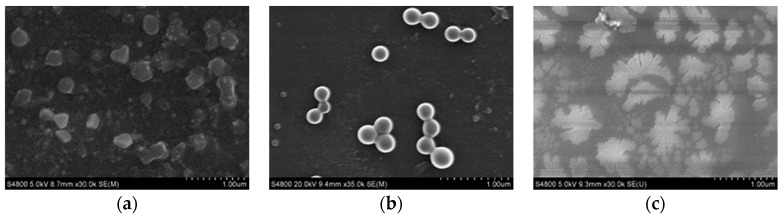
SEM images of SRL nanoparticles prepared at different AS/S ratios: (**a**) AS/S = 10; (**b**) AS/S = 20; (**c**) AS/S = 30.

Solvent/antisolvent (S/AS) flow rate ratio was another crucial factor strongly influencing particle size. After mixing different ratios of drug solution (S) and antisolvent (AS), it was possible to obtain supersaturation, which was the driving force of the precipitation. To investigate the effect of AS/S flow rate ratio, the flow rate of drug solution was fixed at 4.5 mL/min and the flow rates of aqueous solution were varied. As shown in Figure 4, it could be observed the SRL nanoparticle size decreased as the AS/S flow rate ratio increased from 10 to 20. However, when continuing to increase the AS/S FRR from 20 to 30, large flaky drug particles began to appear. This was in agreement with the result reported in our previous work [22].

**Figure 5:**
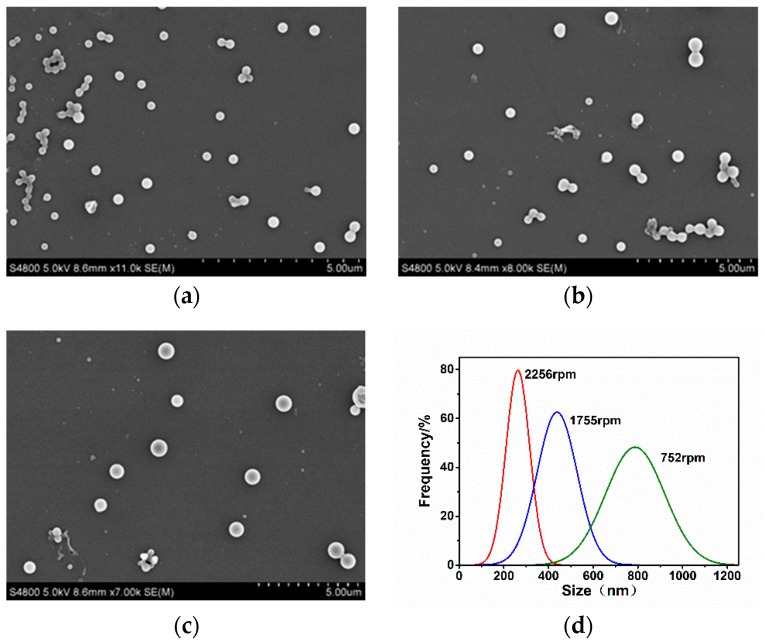
SEM images of SRL nanoparticles prepared under different rotating speeds: (**a**) 37 g; (**b**) 22 g; (**c**) 4 g; (**d**) the corresponding PSDs.

High-gravity field was the most important factor influencing particle size, and was created by the centrifugal force of the RPB, which rotated at a high speed. To facilitate scaled-up production, we enhanced the SRL drug concentration from 10 to 100 mg/mL. Experimental results (Figure 5) showed that the drug particle size decreased with the increase in RPB rotating speed. The rotating speed was one of the key operating parameters of RPB, determining the intensity of micromixing and the rate of mass transfer in the packed bed. As the rotating speed increased, the fluids passing through the packing were broken into nanoscale membranes, silk and drops by the sheer force [25]. As a result, a large and fast-updating phase interface was generated, which tremendously strengthened the mass transfer in RPB [26]. Therefore, the supersaturation in the system was more homogeneous and higher in magnitude, the nucleation rate at various positions turned to more uniform and faster, resulting in a smaller particle size and a narrower PSD.

Additionally, preparing SRL nanocomposite by HGAP process had a higher yield. Under the optimized experimental conditions, the throughput of the anti-solvent and solvent phases reached 5.4 L/h and 0.27 L/h, respectively. About 3.16 kg drug nanocomposite could be produced per day. The RPB showed promising prospects for industrial-scale production, as well as achieving particle size control and good size distribution. The residual solvent test indicated that the as-prepared powder only contained 20 ppm solvent, which satisfies the safety standards of the FDA [27]. Therefore, it is foreseeable that the HGAP process could show huge potential for high-yield preparation of nanodrugs.

### 3.2. Characterization of SRL Nanoparticles

**Figure 6:**
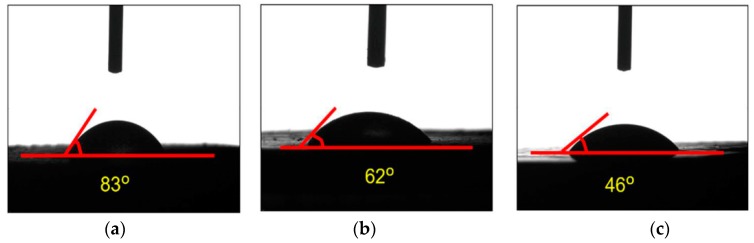
Contact angle studies comparing (**a**) raw SRL; (**b**) physical mixtures; and (**c**) SRL nanoparticle.

As the surface composition was related to the overall hydrophobicity of the powder, it was believed to have a significant impact on the dissolve properties of solid oral drug delivery systems. Several works had researched the critical effect of wettability and contact angles on dissolution rate [28,29,30]. According to one of the studies [31], the contact angles of solid dispersions was related to intrinsic dissolution properties of poorly water-soluble drugs. In addition, the poor wetting properties at large contact angles were considered to be caused by high coverage of hydrophobic surfaces. After loading SRL with PVP-SDS, a large and hydrophilic surface could be framed to reduce the contact angle as shown in Figure 6. The nature of raw SRL was demonstrated to be hydrophobic when its contact angle was 83°. The physical mixture sample showed 21° reduction while that of the SRL nanocomposite showed a 37° reduction compared with the raw SRL. The contact angle results indicated that the nanocrystallization of SRL could change the surface properties of the hydrophobic drug and improve its wettability.

Stability was one of the considerable evaluation indicators for amorphous nanoparticle formulations, since the high-energy solid state was unstable and easily converted into lower-free-energy crystalline forms [32]. Furthermore, the nanoparticles tended to aggregate during storage. For stability studies, the solid dispersions of SRL nanoparticles after 110 days of storage were characterized by FT-IR, XRD, SEM and compared with fresh products.

**Figure 7:**
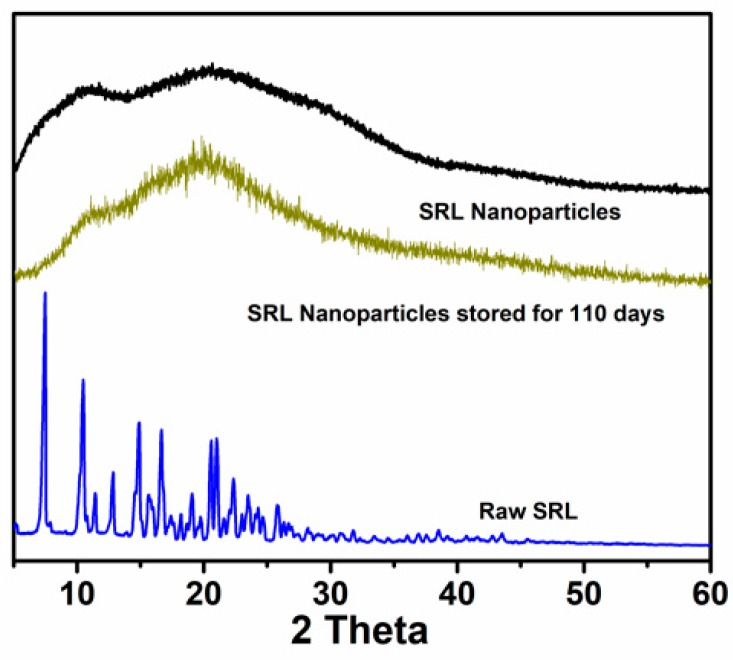
XRD patterns of raw SRL, SRL nanoparticles, and SRL nanoparticles stored for 110 days.

The XRD patterns of SRL samples are shown in Figure 7. It can be observed that the raw sirolimus had sharp crystal diffraction peaks from 5° to 25°, indicating its crystallinity. However, only two broad diffusion peaks appeared in the sample of the nanosized SRL sample, indicating that the SRL nanoparticles in the dispersion were in an amorphous form. After nearly 4 months of storage, the crystal form of the nanoparticles did not change and remained in an amorphous state.

**Figure 8:**
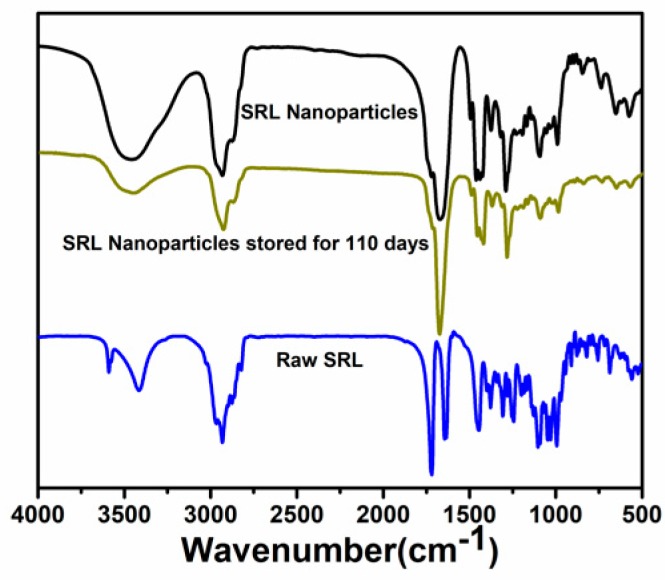
FT-IR spectra of raw SRL, SRL nanoparticles, and SRL nanoparticles stored for 110 days.

The FT-IR test was applied to analyze the chemical composition and molecular structure of the SRL samples. The results are shown in Figure 8. The spectrum of raw SRL was characterized by 3500 cm^−1^ (O–H stretching), 2972 cm^−1^ (C=H stretching), 1680–1640 cm^−1^ (C=C stretching), 1760–1670 cm^−1^ (C=O stretching). In the spectrum of the SRL nanoparticle sample, these characteristic peaks were located at 3500, 2972, 1678 and 1735 cm^−1^, respectively. Changes in the characteristic bands (broadening, frequency shifts and/or disappearance, attenuation) were also detected, which may be due to the crystalline-to-amorphous transition and the loss of crystal water. After storage for nearly 4 months, FT-IR spectra of the nanoparticles was consistent with the freshly prepared sample.

**Figure 9:**
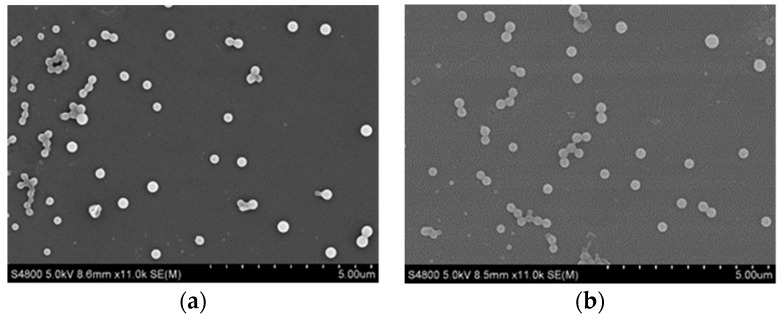
SEM images of (**a**) SRL nanoparticles; (**b**) SRL nanoparticles stored after 110 days.

SEM images (Figure 9) showed that the SRL nanoparticles still maintained their original spherical shape, good dispersion and particle size at 250 nm after being stored 110 days. All of these indicate that the SRL nanoparticles exhibited excellent physical stability and could be post-processed as tablets.

### 3.3. Dissolution Test

**Figure 10:**
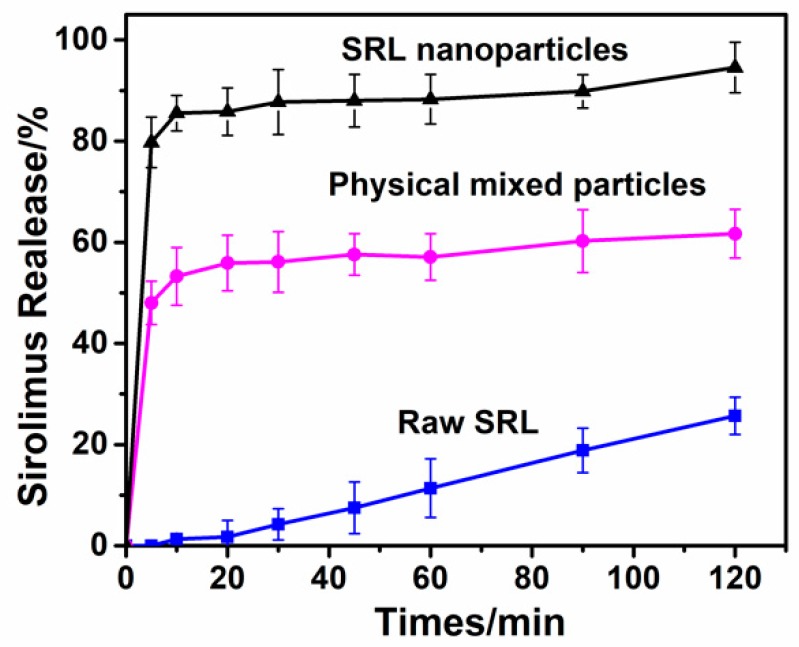
Dissolution profiles of raw SRL, physical mixed particles and SRL nanoparticles.

The bioavailability of drugs is an important evaluation index of solid pharmaceutical preparations. In vitro dissolution testing based on the theory of the BCS (biopharmaceutical classification system) is the best alternative to an in vivo bioequivalence study [33]. The dissolution tests were performed at 37 °C, phosphate buffer saline (PBS) solution (pH 6.8) with 0.5% SDS was used as the dissolution medium. Figure 10 illustrated the dissolution profiles of Raw SRL, SRL nanoparticles and physical mixed particles. SRL nanoparticles exhibited a high burst release in the first 5 min—about 80% of the drug was released—while the physical mixed particles had only released 60% after 120 min. In comparison, raw SRL was almost not released at all in the same period, and only 23% had been released after 120 min. The significant increase in the solubility of the nanoparticles could be attributed primarily to the small particle size and amorphous nature.

### 3.4. Dissolution Test Tablet Formulation

Excipients and formulation should be optimized to meet the ideal properties and better performance of in vitro dissolution. We chose microcrystalline cellulose as diluent, mannitol as filler, crosslinked polyvinylpyrrolidone as disintegrants and magnesium stearate as lubricants. Tablets with nanosized SRL were prepared and tested with the tablet appearance, hardness, friability and time of disintegration. Table 1 shows the properties of tablets which compressed under the pressure 2.5–15 MPa. It can be observed that with the increase in compression force, the friability decreases and the hardness and disintegration time increase.

**Figure 11:**
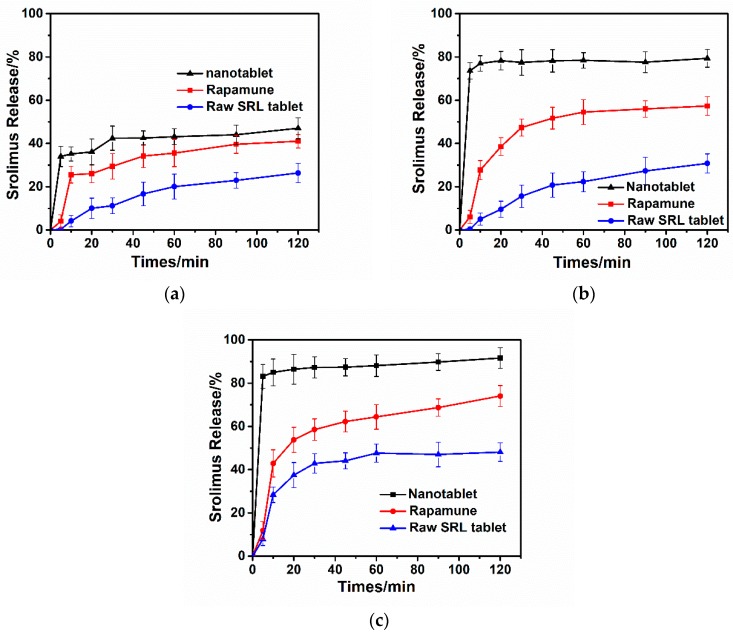
Dissolution profiles of nanotablet, Rapamune^®^ and raw SRL tablet (**a**) pH = 1.2; (**b**) pH = 4.5; (**c**) pH = 6.8.

Figure 11 compares the in vitro release profiles of different tablets. The incorporated amount of drug in the tablet was 1 mg, which is the same as the pharmacological dose of marketed tablets. As a control group, the same excipient formula was used for raw SRL tablets. Dissolution results showed that in different simulated gastrointestinal pH environments, the dissolution rate of nanotablets was much higher than that of commercial tablets and raw SRL tablets. Drug release was extremely fast in the simulated intestinal pH of 6.8, reaching 80% drug within 5 min.

**Figure 12:**
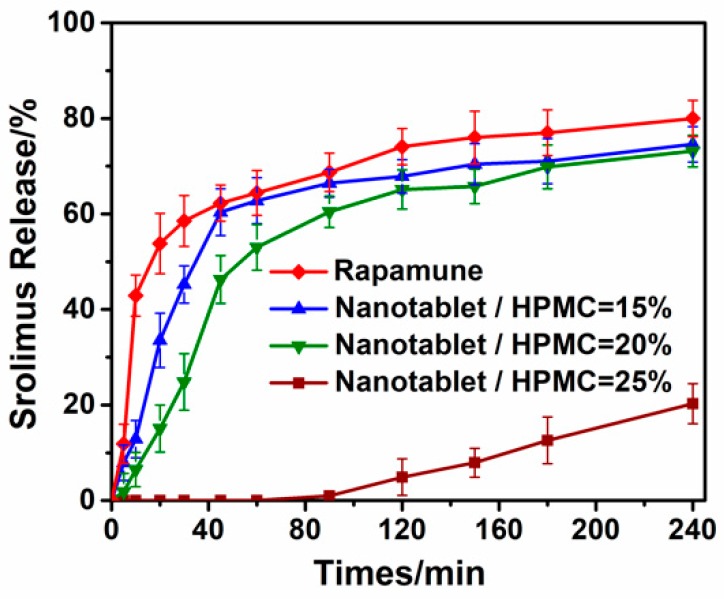
Effect of HPMC amount on dissolution of tablets.

Compared to the commercial tablet, the nanotablet exhibited a drug instant release condition in the first 5 min. An attempt was made to avoid this circumstance by adding hydroxypropyl methylcellulose (HPMC) to the tablets. We found that HPMC had a significant influence on drug release, as shown in Figure 12. The dissolution rate of the drug dropped as the HPMC dosage increased. This was mainly due to the fact that HPMC could quickly wet-out in water, swelling to form a gel layer, and had an excellent sustained release capacity for a variety of different types of drugs [34]. When the content of HPMC was 15 wt. %, the dissolution tendency of SRL nanotablets was close to that of marketed tablets, but the dissolution rate in the first 45 min was slower than that of marketed tablets.

**Figure 13:**
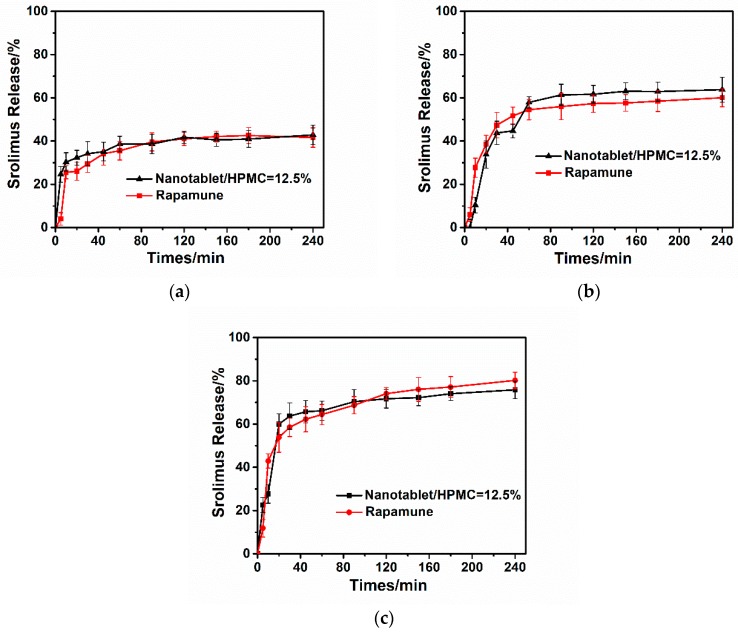
Dissolution profiles of optimized nanotablets and Rapamune^®^ (**a**) pH = 1.2; (**b**) pH = 4.5; (**c**) pH = 6.8.

To control the dissolution behavior of the nanotablets to be consistent with the commercially available tablets to ensure the same bioequivalence, we adjusted the dosage of HPMC to 12.5% in the formula. As shown in Figure 13, the dissolution behavior of the nanotablets was close to the marketed tablets under different simulated gastrointestinal pH conditions. Under simulated intestinal pH conditions, 23% of the drug was released within 5 min, it reached 60% at 20 min, and then it maintained stable release till 4 h, indicating that the in vitro dissolution performance of optimized tablet was almost the same as that of Rapamune^®^.

## 4. Conclusions

In summary, stable amorphous SRL nanoparticles with a mean size of 250 nm were successfully produced by a high-throughput method using high-gravity technology. The particle size could be controlled by adjusting the operating conditions. As-prepared SRL nanoparticles were both chemically and physically stable for 4-month storage and were further post-processed as tablets. The tablets with SRL nanoparticles dissolved more rapidly than commercial tables under simulated gastrointestinal pH conditions. Moreover, dissolution behavior of the tablets could be manipulated to imitate Rapamune^®^, a commercial product produced using a top-down method via optimizing the formulation to realize controlled drug release. These results suggest that high-gravity technology should be a new strategy for nanosized dosage forms.

## Figures and Tables

**Table 1:** Technological characterization of tablets, *n* = 6.

Batch	Compression Force (MPa)	Mean Weight (mg)	Hardness (N)	Friability (%)	Disintegration Time (min)
1	2.5 ± 0.5	320 ± 4.6	31.7 ± 1.3	0.869 ± 0.005	13 ± 1.5
2	5 ± 0.8	312 ± 2.2	37.5 ± 2.5	0.557 ± 0.009	18 ± 2.6
3	10 ± 1.2	316 ± 5.7	43.2 ± 2.1	0.524 ± 0.011	27 ± 2.1
4	15 ± 0.9	321 ± 3.2	52 ± 1.8	0.483 ± 0.019	46 ± 1.6
